# AprGPD: the apricot genomic and phenotypic database

**DOI:** 10.1186/s13007-021-00797-4

**Published:** 2021-09-23

**Authors:** Chen Chen, Huimin Liu, Ningning Gou, Mengzhen Huang, Wanyu Xu, Xuchun Zhu, Mingyu Yin, Haikun Bai, Lin Wang, Ta-na Wuyun

**Affiliations:** 1 State Key Laboratory of Tree Genetics and Breeding, Non-Timber Forest Research and Development Center, Chinese Academy of Forestry, Zhengzhou, China; 2 Kernel-Apricot Engineering and Technology Research Center of State Forestry and Grassland Administration, Zhengzhou, China; 3 Key Laboratory of Non-Timber Forest Germplasm Enhancement and Utilization of National Forestry and Grassland Administration, Zhengzhou, China

**Keywords:** Apricot, AprGPD, Genome, Phenotype, Transcription factors, Variation, Expression

## Abstract

**Background:**

Apricot is cultivated worldwide because of its high nutritive content and strong adaptability. Its flesh is delicious and has a unique and pleasant aroma. Apricot kernel is also consumed as nuts. The genome of apricot has been sequenced, and the transcriptome, resequencing, and phenotype data have been increasely generated. However, with the emergence of new information, the data are expected to integrate, and disseminate.

**Results:**

To better manage the continuous addition of new data and increase convenience, we constructed the apricot genomic and phenotypic database (AprGPD, http://apricotgpd.com). At present, AprGPD contains three reference genomes, 1692 germplasms, 306 genome resequencing data, 90 RNA sequencing data. A set of user-friendly query, analysis, and visualization tools have been implemented in AprGPD. We have also performed a detailed analysis of 59 transcription factor families for the three genomes of apricot.

**Conclusion:**

Six modules are displayed in AprGPD, including species, germplasm, genome, variation, product, tools. The data integrated by AprGPD will be helpful for the molecular breeding of apricot.

**Supplementary Information:**

The online version contains supplementary material available at 10.1186/s13007-021-00797-4.

## Background

Apricots, belonging to the Rosaceae family and section Armeniaca (Lam.) Koch [1], are cultivated worldwide and considered one of the most delicious temperate tree fruits [2]. Apricot has a long history of cultivation in China[3]. A total of ten species belongs to the Armeniaca section, among these species, nine species, including *Prunus armeniaca*, *P. sibirica*,* P. mandshurica*,* P. holosericeae*,* Prunus* × *dasycarpa*,* P. zhidanensis, P. zhengheeniss*,* P. limeixing*, *and P. mume*, are native to China [3–5], whereas, *P. brigantina* is endemic in French Alps [6].

The modern sequencing technology has boosted the genome and transcriptome data of apricot and the phenotypic data of rich germplasm resources in apricot have been accumulated. Therefore, a comprehensive database is needed to construct for integrating these data, which is convenience for researchers and breeders of apricot and other species in Rosaceae. Here, we have summarized three high-quality and chromosome scale assemblies of genomes in AprGPD, including *P. sibirica* (F106, 219 Mb with a contig N50 length of 6.70 Mb, eight chromosomes), *P. armeniaca* (Sungold, 217 Mb with a contig N50 length of 7.13 Mb, eight chromosomes), and kernel consumption apricot (Longwangmao, 225 Mb with a contig N50 length of 6.91 Mb, eight chromosomes), the reference genome was version 0.9 for all species. AprGPD includes 90 RNA sequencing (RNA-seq) data, 306 genome resquencing data, and 1692 germplasms (58 phenotypic types). All data included in the AprGPD were collected by the authors. Based on these data, we have conducted gene annotation, transcriptome analysis, detailed analysis of transcription factor (TF) family, annotation of variation sites, construction of the metabolic pathways, classification of quantitative characteristics, and prepared figures for the visual representation of the phenotypic data. AprGPD also offers various query, analysis, and visualization tools. A total of six modules are displayed in AprGPD, including species, germplasm, genome, variation, product, and tools. This database is a useful resource for the  apricot research.

## Construction and content

The data of AprGPD are stored in MySQL database on a Ubuntu server. User-friendly interfaces are developed using JavaScript, HTML5, and CSS3. Query searches are achieved suing JavaScript and PHP. AprGPD is divided into six modules base on different data and applications. The data and functions of each module are described as follow:

### Species module

**Fig. 1:**
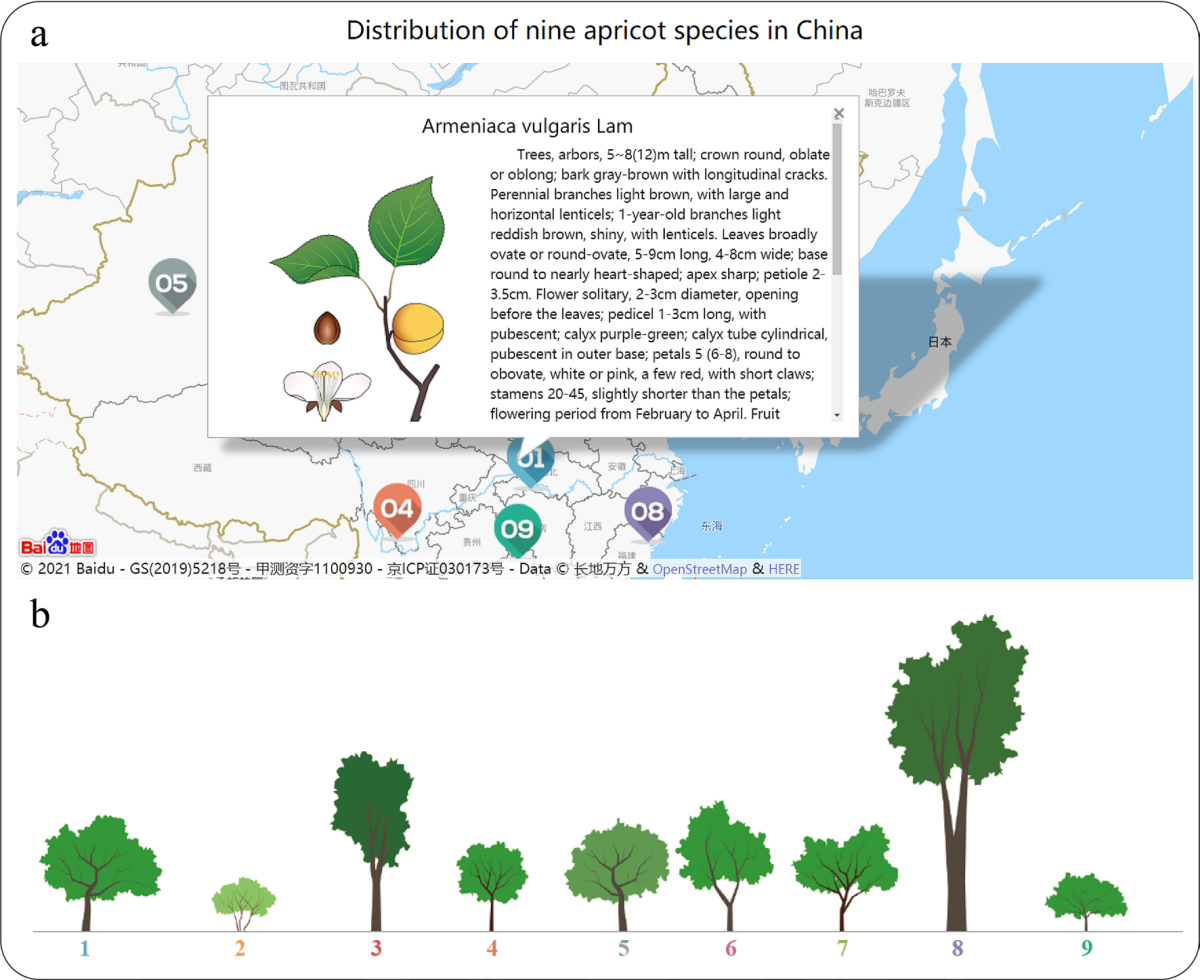
Nine species of apricot from China. 01–09 represent nine species. **a** The distributions, descriptions, and pictures of the nine species. **b** The tree type and relative height of nine species

The species module exhibits the distribution and phenotypic traits, including flower, leaf, fruit, seed, and tree type of the nine species from Armeniaca section in China (Additional file 1: Table S1) and provides a visual representation of each species (Fig. 1).

### Germplasm module

**Fig. 2:**
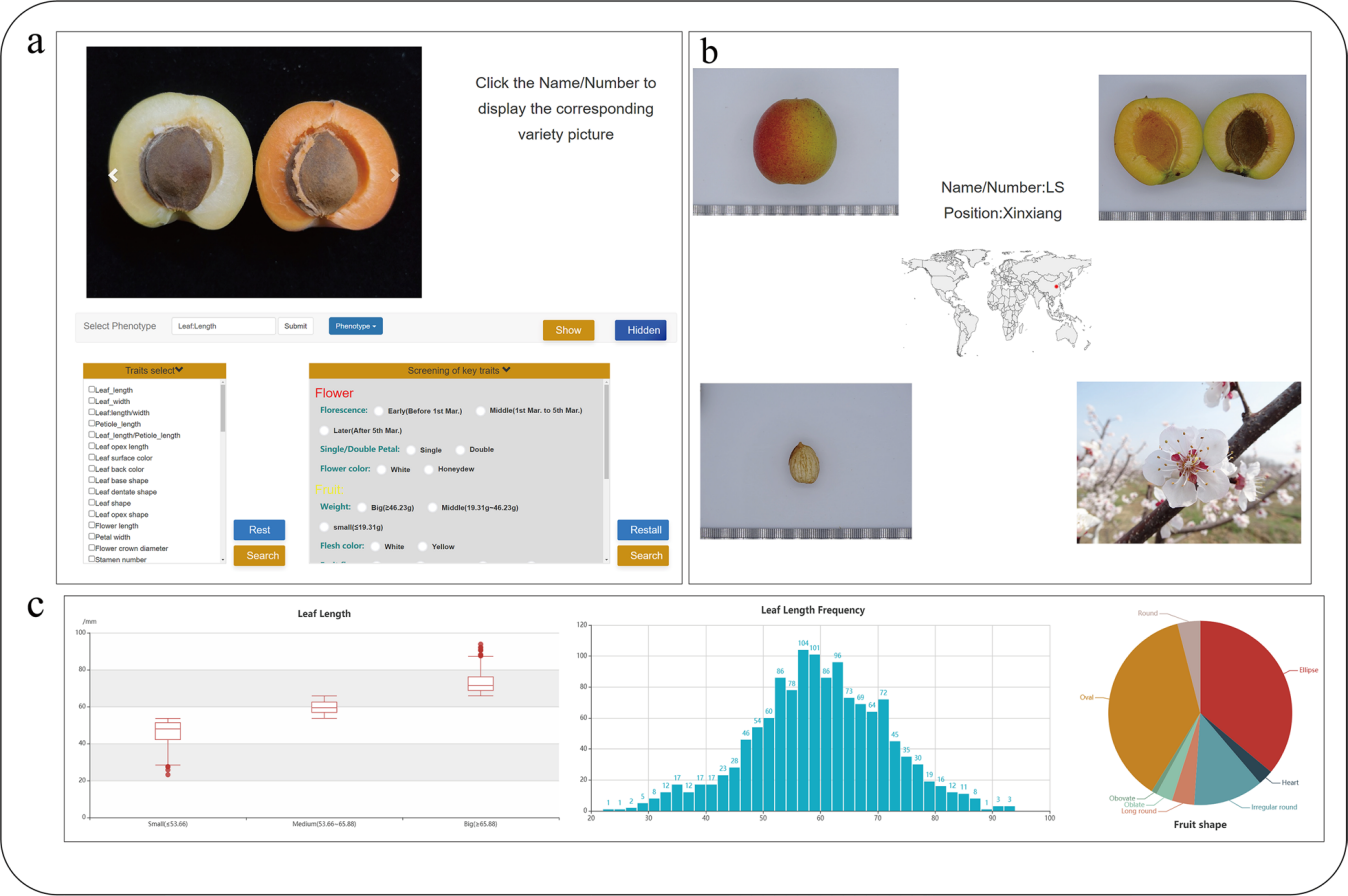
Germplasm module. **a** Search tools; **b** Images and distribution of the sample; **c** Statistical chart of quantitative (frequency chart and boxplot) and qualitative traits (pie-chart)

The germplasm module contains 58 phenotypic data types of 1692 germplasms, and clicking on the corresponding name, then, displaying pictures of their flowers, fruits, seeds, and distribution information (Fig. 2b). The intersting traits can also be quickly observed (Fig. 2a). The pie chart provides statistical information on qualitative traits, and the frequency chart provides the overall summary of quantitative traits (Fig. 2c). We divided the quantitative traits into three levels by using  μ± 0.5246δ for data showing normal distribution (μ and δ indicated mean and standard deviation, respectively) and $y = G \pm \left( {\text{1/2 + n}} \right)x$ (*n* = 0, 1, 2, 3, 4; *G* is the median of the data, and *x* is the grade difference) for data showing non-normal distribution as previous descriptions [7, 8].

### Genome module

The genome module is divided into five submodules, including gene annotation, metabolic pathway, gene family, gene network, and transcription factors. For each submodule, the data and basic search functions have been described.

#### Gene annotation

**Fig. 3:**
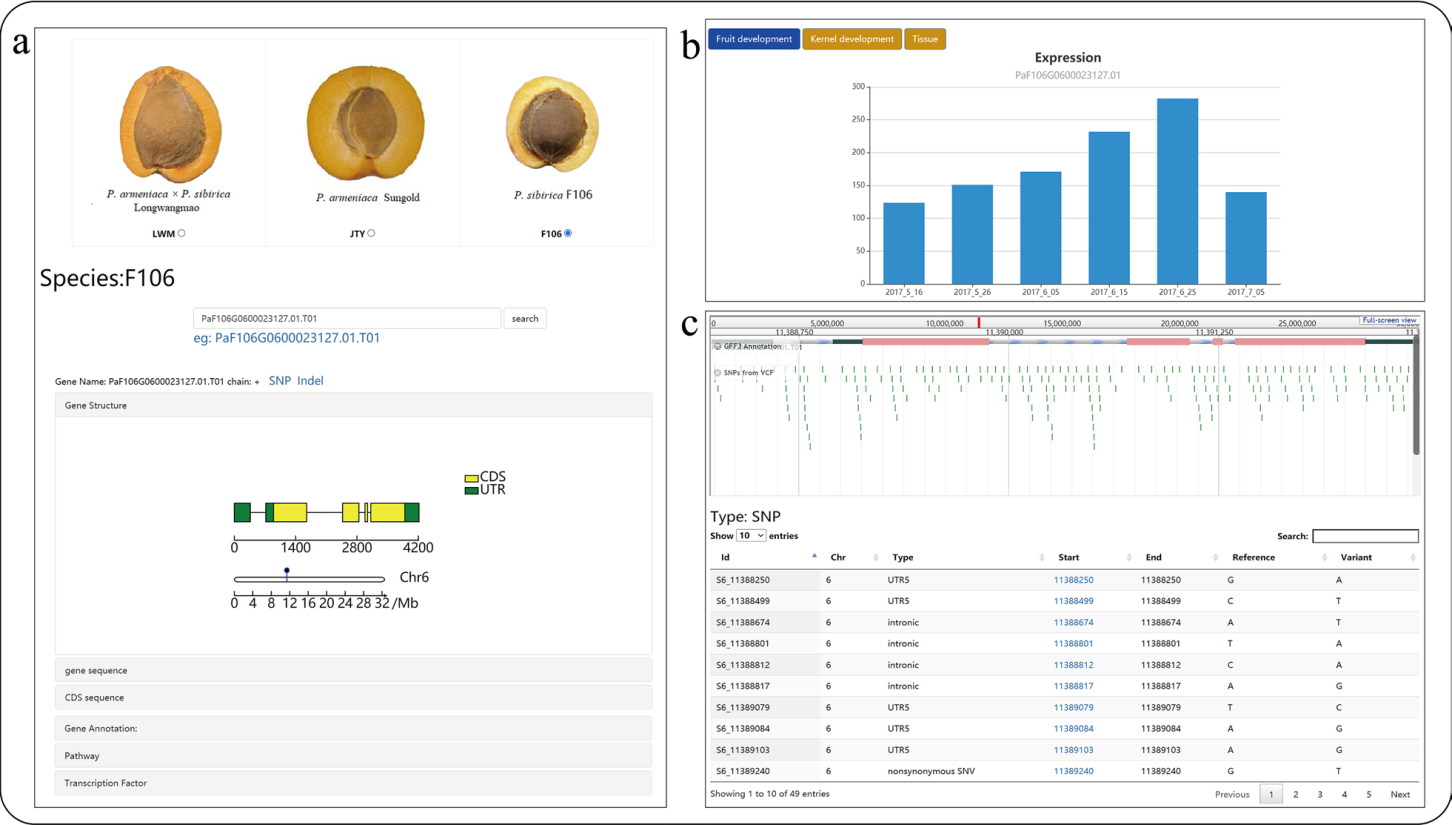
Gene annotation submodule. **a** Gene annotation information; **b** Gene expression; **c** Gene variation information

A total of 98,615 protein-coding genes were predicted from these three genome assemblies, which included 32,959 genes from *P. sibirica* (F106), 32,669 genes from *P. armeniaca* (Sungold), and 32,987 genes from *P. armeniaca* × *P. sibirica* (Longwangmao). The information of gene structure, gene annotations from Gene Ontology (GO) and KEGG annotation (Fig. 3a), gene expression profiles (Fig. 3b), and variations are displayed interactively (Fig. 3c) in this submodule. Other submodules, including metabolic pathways, transcription factors, gene network, and gene family, are integrated with gene annotation.

#### Metabolic pathways

**Fig. 4:**
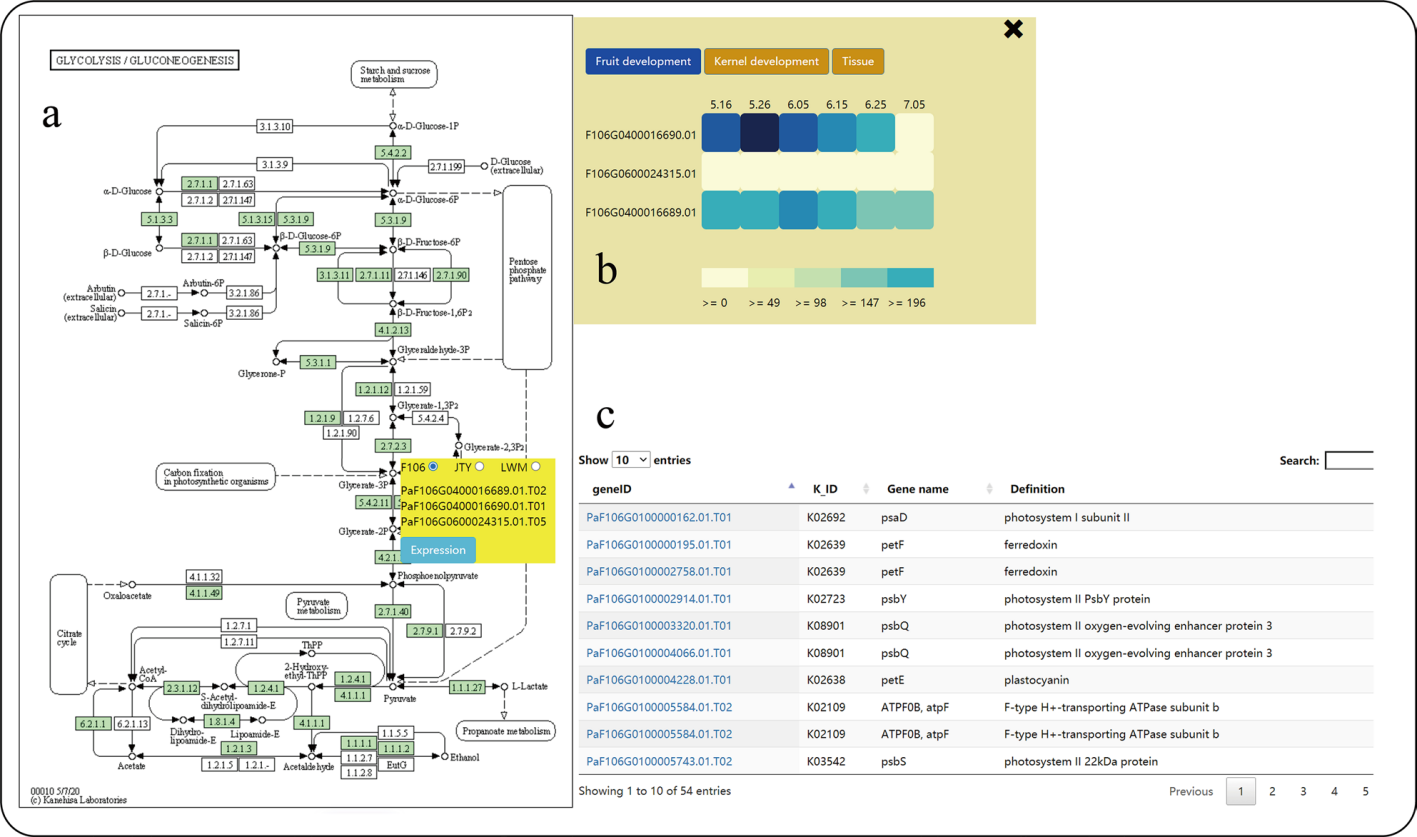
Metabolic pathway submodule. **a** KEGG pathway, demo of Ko 00010; **b** The expression pattern of candidate genes; **c** The detailed information of genes in the pathway

KEGG pathway maps are the graphical representations of the reaction networks, and each map is  summarized by experimental evidence from the literature [9]. We have obtained the KEGG orthologs for the Sungold, F106, and Longwangmao genomes and generated their metabolic pathways. Moreover, we have constructed five additional pathways (Additional file 1: Figure S1) related to the flowering [10]. When the cursor of the mouse is moved to the green nodes, all the gene IDs corresponding to the enzymes are displayed, and the expression of the genes for fruit development, kernel development, and different tissues can be observed conveniently. Each pathway is displayed on a separate web page, and detailed information of all the genes in this pathway is displayed in a tabular form at the bottom of the page (Fig. 4).

#### Gene family

**Fig. 5:**
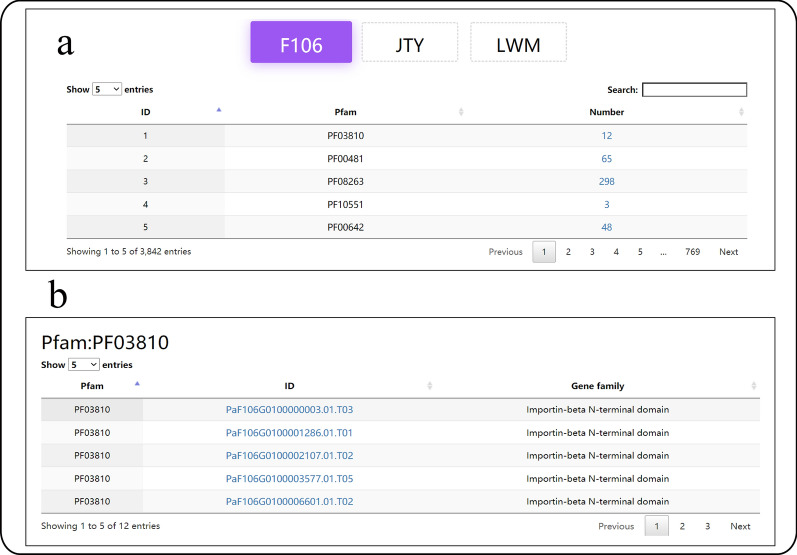
Gene family submodule. **a** Information about the number of each gene family; **b** Details about the gene family members

Based on the HMM Pfam, we have summarized the protein domain families (gene families) and established tables for the ease of querying and viewing (Fig. 5). In total, 63,121 genes, including Longwangmao (21,012), Sungold (21,444), and F106 (20,665) are assigned to 3842 gene families.

#### Gene network

**Fig. 6:**
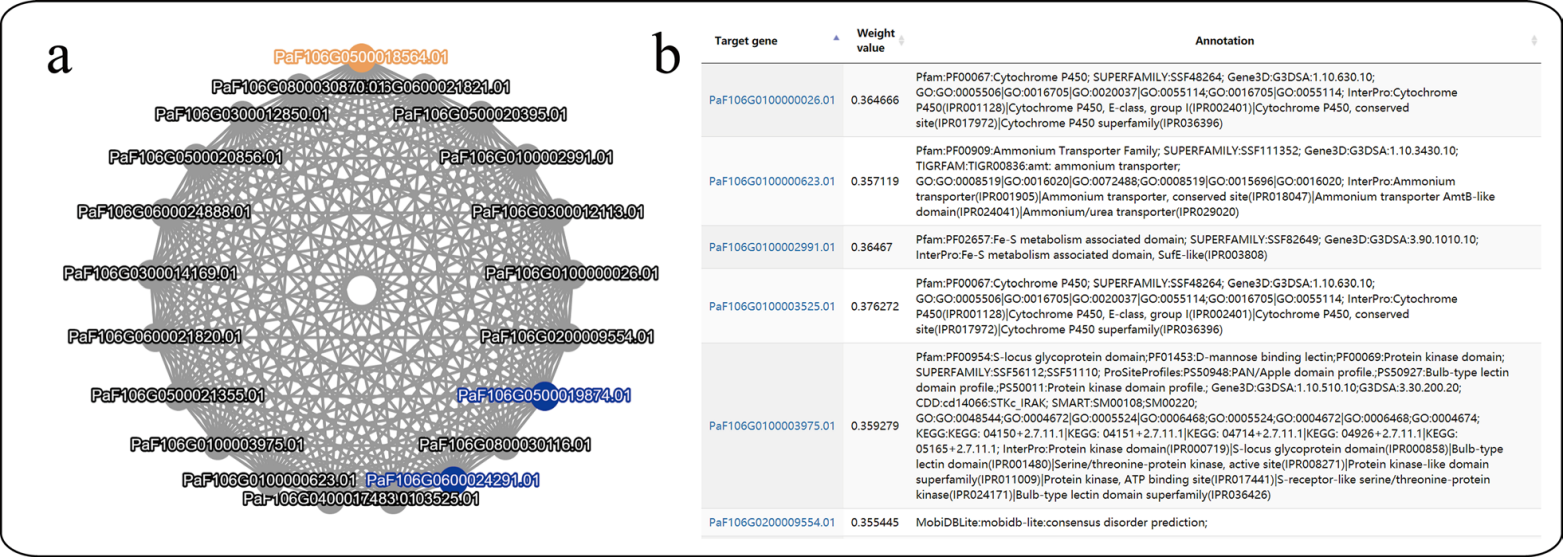
Co-expression of genes. **a** Network: transcription factors (blue) and target gene ID (yellow); **b** Annotation of the co-expressed genes

We have collected 90 RNA-seq samples for F106 (30), Sungold (22), and Longwangmao (38), including fruit, kernel, leaves, flower, and flower bud at different stages. The expression pattern of each gene is calculated and normalized to fragments per kilobase of transcript per million mapped fragments (FPKM). Based on the FPKM values, the co-expression network is constructed by the WGCNA[11] (version 1.0) package in R (weight value > 0.15) (Fig. 6).

#### Transcription factors

Transcription factors are important regulators of plant growth, development, and external stress. A total of 59 transcription factor (TF) families have been analyzed in detail at the whole-genome level of three apricot genomes. TFs are determined by HMMER [12] (version 3.0) and iTak [13]. The presence of the TF domain is further confirmed using SMART [14] and CDD [15]. Multiple sequences alignment are performed using ClustalX [16] (version 2.1), and phylogenetic trees are constructed using MEGA (version X) [17]. MEME Suite [18] is used to determine motifs in transcription factor protein sequences, where the width of the motif is 6–200, and the maximum number of motifs is 20. Syntenic blocks are inferred using MCScanX [19]. Gene structure, chromosome location, and collinearity are visualized using TBtools (version 1.089) [20]. Phylogenetic trees are aesthetically improved using iTol [21]. Additionally, the links are established with PlantTFDB [22] to provide users with valuable information.

**Fig. 7:**
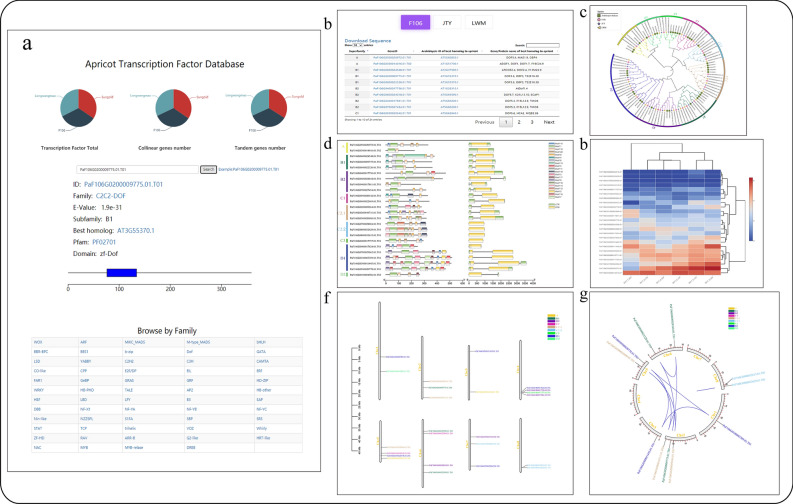
Information of transcription factors in apricot. **a** Query tool; **b** Information of TFs; **c** Phylogenetic tree; **d** Gene structure and motif distribution; **e** Expression heatmap; **f** Distribution of TFs on eight chromosomes; **g** Syntenic relationships

Users can view the respective TF page or find more detailed information using the query function (Fig. 7a). The valuable information is collected through transcription factor analysis, including homology alignment (Fig. 7b), comparison among the three genomes (Fig. 7b, c), subfamily classification (Fig. 7c), domains (Fig. 7d), gene expression (Fig. 7e), chromosome location (Fig. 7f), and collinearity results (Fig. 7g).

### Variation module

**Fig. 8:**
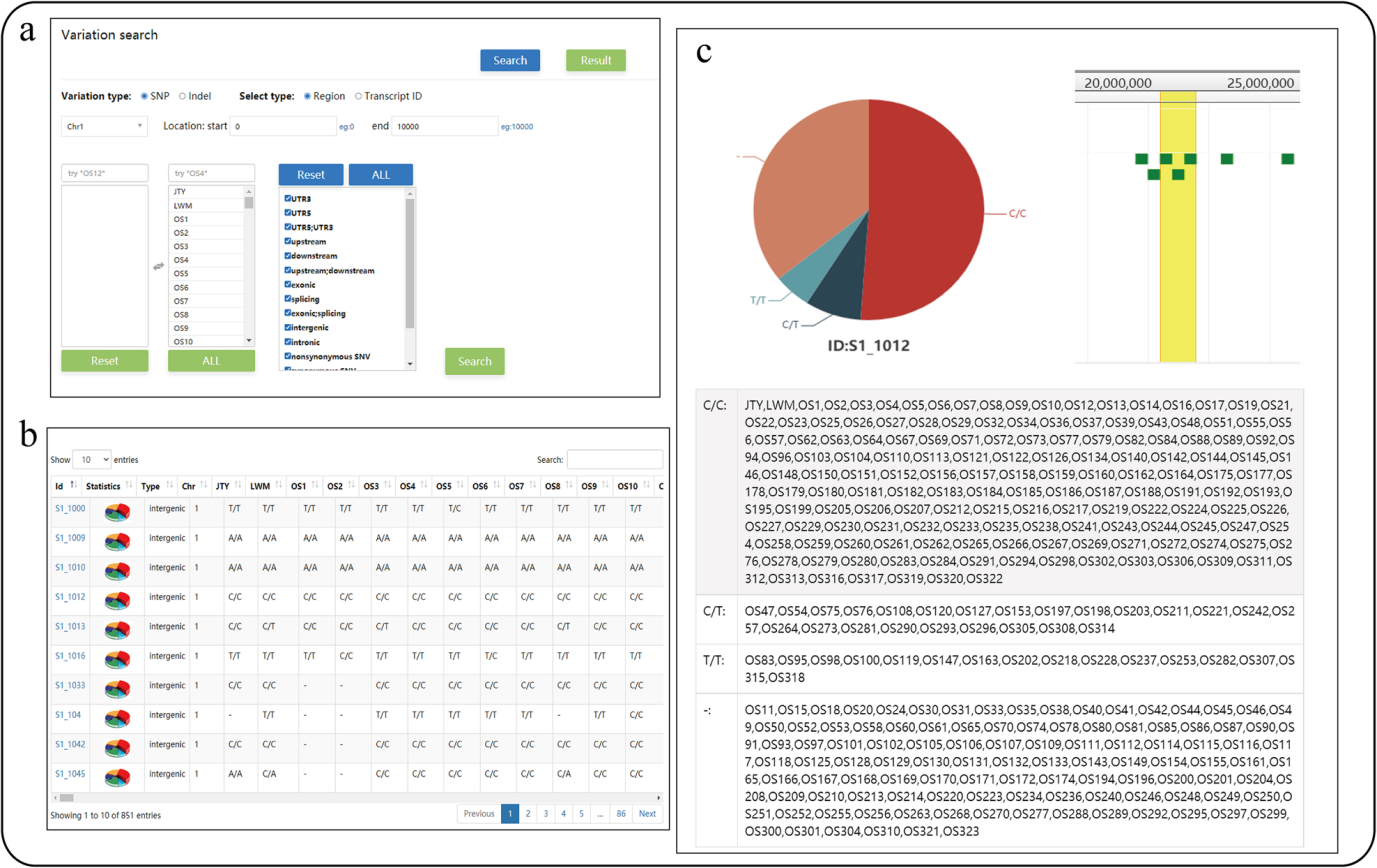
Search and display information on variations. **a** Comparative search tool; **b** Variation information displayed in a table; **c** Statistical information on variation displayed using JBrowse, pie charts, and tables

The single-nucleotide polymorphisms (SNPs) and insertions-deletions (Indels) of 306 accessions have been collected from our previous study, and minor allele frequencies over 0.05 of variants have been filtered out using plink [23] (version 1.9); all filtered nucleotide variants are annotated using ANNOVAR [24]. Annotation information of 8,838,420 (SNPs) and 1,650,013 (indels) is obtained. The information on the variation of each sample, including variation in positions and allele types, is sorted, and a comparative search tool is established (Fig. 8a), which can be searched by gene ID or locations; the information on variations are displayed in tabular form (Fig. 8b). Variations can also be browsed interactively through JBrowse [25] by clicking on the variation ID (Fig. 8c). Statistical information on variation is provided through pie charts and tables, which can be accessed by clicking the pie chart icon (Fig. 7c). In addition, The information about structural variations (SVs, ≥50 bp) was obtained through a comparison among the three genomes. In total, 2306 insertions (843, 721, and 742 for F106 vs. Sungold, F106 vs. Longwangmao, and Sungold vs. Longwangmao, respectively) and 1296 deletions (427, 409, and 460 for F106 vs. Sungold, F106 vs. Longwangmao, and Sungold vs. Longwangmao) were obtained, which contain 2888 genes.

### Product module

**Fig. 9:**
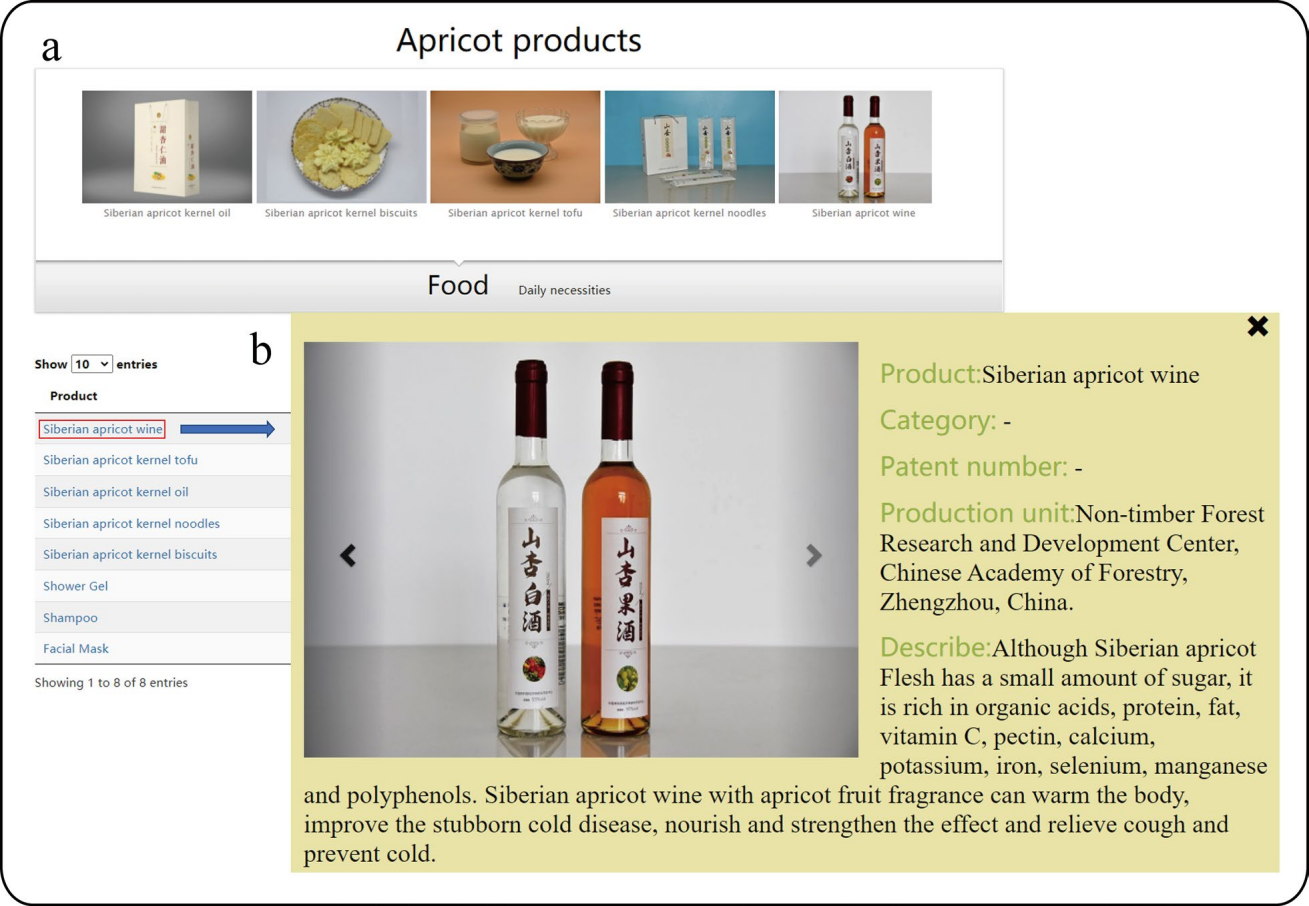
The products involved in apricot. **a** The product image. **b** Details of corresponding apricot-derived product

Apricot has high nutritional and commercial value [2]. Apricot kernel is rich in protein, crude fat, calcium, phosphorus, and iron, and has good nutritional and health effects. The apricot fruit has a pleasant aroma and is rich in nutrients such as sugar, vitamin C, and carotenes. We have developed some products of apricot, including foods and cosmetics. The edible products of apricot include apricot wine, apricot kernel oil [26], apricot kernel tofu [27], apricot kernel noodles, and apricot kernel biscuits. The use of apricot in cosmetics includes facial masks, essential oils, shampoos, and shower gels. The detailed information of all products, such as product image, patent number, etc., are displayed in this module by clicking on the product name (Fig. 9).

### Tools module

The **BLAST** program, developed by ViroBLAST [28], provides more BLAST options to obtain specific information from the gene, CDS, and protein databases of all reference genomes. Information on genomes (Sungold, Longwangmao, and F106) and the variations of 306 accessions (reference F106) were visualized by **JBrowse** [25]. Bud dormancy plays an important role in fruit yield. The chilling requirement (CR) is the temperature required for deciduous fruit trees to release their natural dormancy and is an important quantitative trait for measuring dormant release. We developed a tool for calculating chilling requirements (**CHR**) for the convenience of the researchers; the tool contains 0–7.2 °C, Utah, and dynamic models. The CHR tool is developed by using PyQt5 (https://github.com/Chilling-requirements/Chilling-software/releases), which also had more functions, such as, the custom model and the model of growing degree hours for heart requirements. **Expression visualization** [29] could help users quickly observe the expression patterns of the related genes. The results of the synteny analysis with three pairs of reference genomes (Sungold vs. F106, Sungold vs. Longwangmao, and F106 vs. Longwangmao) are visualized by **SynVisio** (https://github.com/kiranbandi/synvisio).

## Utility and discussion

To provide more convenience for users, the query tools in each module are constructed. Users could be obtain information, including phenotype, gene annotation, transcription factors, metabolic pathways, co-expression network, variation, by query tools. The help module provides the detailed of user tutorial information. To make the AprGPD even better to understand, several demonstrations have been provided below.

### Species and phenotype query

If users are interested in species information, they can click on the species number. For example, a user can click on No. 4 to retrieve information and images of *P. holosericeae*. The germplasm module addresses any query on phenotype data. Boxplots were used to show three levels of quantitative traits. The Pie-charts and frequency charts were used to illustrate the distribution of quantitative and qualitative traits, respectively. For example, users could click on the name of Gongfuoxing to show its image and location information, and also could obtain the overall information on kernel dry weight using frequency charts. All quantitative traits are divided into three grades, if users want to get samples with larger kernel dry weight, they could select the maximum level (≥ 0.39 g) to filter the samples.

### Gene query

Abundant query functions could help users quickly find the information of related genes. For example, the information on PaF106G0500018564.01 needs to be obtained. First, the user inserts the gene ID in the annotation module and clicks on the search button. The expression level of the gene is showen by histogram, and the highest expression at the end of fruit development, and gradually decreased and then increased during kernel development were displayed. Second, the user inserts the gene ID in Pathway module and finds that it is not annotated to the metabolic pathway. Third, the user searches for this gene in the transcription factor module and finds that belongs to the subfamily of the WOX family. Fourth, the user searches for this gene in the Gene network module and sets the weight at 0.35, and two co-expression genes (*WRKY,* PaF106G0500019874.01 and PaF106G0600024291.01.T01) are showed. Fifth, the user searches and observes the information of variation in this gene using the comparative search tool of the variation module and finds that PaF106G0500018564.01 has 20 SNPs and 9 INDELs.

### Transcription factor family query

The detailed analysis of transcription factors are of great use to researchers. An example of MIKC_MADS is illustrated. First, the user could be find the number of members in the MIKC_MADS family page. Second, comparative analysis of all MIKC_MADS family genes in three species were shown by using a phylogenetic tree (Additional file 1: Figure S2) and table, a total of 14 subfamilies are divided. Third, the distributions of conserved domains of MIKC_MADS family genes were obtained (Additional file 1: Figures S3–5). For example, SOC1 contains motif17, SVP contains motif5 and motif6. Fourth, AP3, SVP, AGL6, SOC1, AGL9, and AP1/FUL members have fragment duplication by chromosomal collinearity (Additional file 1: Figures S6–8).

The heatmap of related genes were displayed by expression visualization in tool (Additional file 1: Figures S9–11). For example, *SVPs* (PaF106G0100006115.01.T01, PaF106G0100006117.01.T02, PaF106G0100006114.01.T06, PaLWMG0100006313.01.T01, PaLWMG0100006315.01.T02, PaLWMG0100006312.01.T02) shows an increasing trend during kernel development, whereas, *AGL9s* (PaF106G0100002725.01.T01, PaF106G0300011661.01.T03, PaLWMG0100002950.01.T04) show a gradually decrease during kernel development.

### Value and future directions

At present, AprGPD contains the phenotypic, genome, transcriptome data, and variation information, and a series of analysis base on these data, which provides more valuable information to aptivot research. We completed a detailed analysis of 58 transcription factor families from three genomes in AprGPD, which allows comparisons in various apricots and in plant species of Rosaceae. We developed a calculation tool of chilling requirements, which provids convenience for researchers. In the future, we will be further the new genome, transcriptome, and phenotype data of apricot, establish, and improve the multi-omics analysis platform.

## Conclusions

In this study, the database of apricot genome and phenotype was established, which provides a database of comprehensive phenotype, genome, and transcriptome resources of apricots. AprGPD is composed of six modules, including genome, predicted genes and proteins, functional annotations and gene expression profiles. In addition, this database also providess various query, analysis, and visualization tools. Our AprGPD will become a active platform for researchers and breeders of apricot.

## Supplementary Information

**Additional file 1: Table S1.** Distribution of nine species in China. **Figure S1.** Pathways involved in flowering time. **Figure S2.** Phylogenetic tree of MIKC_MADS family. **Figure S3.** Gene structure and conserved motifs of MIKC_MADS family in *P. sibirica* (F106). **Figure S4.** Gene structure and conserved motifs of MIKC_MADS family in *P. armeniaca* (Sungold). **Figure S5.** Gene structure and conserved motifs of MIKC_MADS family in *P. armeniaca* × *P. sibirica* (Longwangmao). **Figure S6.** Chromosomal collinear of MIKC_MADS family in *P. sibirica* (F106). **Figure S7.** Chromosomal collinear of MIKC_MADS family in *P. armeniaca* (Sungold). **Figure S8.** Chromosomal collinear of MIKC_MADS family in *P. armeniaca* × *P. sibirica* (Longwangmao). **Figure S9.** Expression of MIKC_MADS family in *P. sibirica* (F106). (a) Expression of fruit development. (b) Expression of kernel development. **Figure S10.** Expression of fruit development of MIKC_MADS family in *P. armeniaca* (Sungold). **Figure S11.** Expression of MIKC_MADS family in *P. armeniaca* × *P. sibirica* (Longwangmao). (a) Expression of fruit development. (b) Expression of kernel development.

## Data Availability

AprGPD is freely available at the following address: http://apricotgpd.com. All functionalities of AprGPD have been tested in Google Chrome, 360, QQ, and Safari Browser.

## References

[1] Rehder A (1927). Manual of cultivated trees and shrubs hardy in North America. Taxon.

[2] Kafkaletou M, Kalantzis I, Karantzi A, Christopoulos MV, Tsantili E (2019). Phytochemical characterization in traditional and modern apricot (*Prunus armeniaca* L.) cultivars—nutritional value and its relation to origin. Sci Horticult.

[3] Zhebentyayeva TN, Ledbetter C, Burgos L, Llácer G (2012). Fruit breeding. Handbook of plant breeding.

[4] Zhang JT, Zhang J (2003). Chinese fruit tree: apricot.

[5] Zhang QP, Liu WS, Ning L, Zhang YP, Ming X (2017). Allelic variation of simple sequence repeats markers linked to PPV resistance in Chinese apricot. Hortic Sci.

[6] Liu S, Decroocq S, Harte E, Tricon D, Decroocq V (2020). Genetic diversity and population structure analyses in the Alpine plum (*Prunus brigantina* Vill.) confirm its affiliation to the Armeniaca section. Tree Genet Genomes.

[7] Wang YZ, Sun H, Li Y, Zhang J (2008). Classification criteria of some quantitative characteristics of apricot germplasm resources. Chin Agric Sci Bull.

[8] Zhou H, Wang F, Jiang Z, Hao C, Wang W, Liu T (2015). Studies on the classify standard for quantitative characters of sorghum DUS testing in Jilin province I. Measurement of single characters. Jilin Agric Sci.

[9] Minoru K, Susumu G, Yoko S, Miho F, Mao T (2012). KEGG for integration and interpretation of large-scale molecular datasets. Nucleic Acids Res.

[10] Srikanth A, Schmid M (2011). Regulation of flowering time:all roads lead to Rome. Cell Mol Life Sci.

[11] Langfelder P, Horvath S (2008). WGCNA: an R package for weighted correlation network analysis. BMC Bioinform.

[12] Eddy SR (2009). A new generation of homology search tools based on probabilistic inference. Genome Inf.

[13] Yi Z, Chen J, Sun H, Rosli HG, Pombo MA, Zhang P, Banf M (2016). iTAK: a program for genome-wide prediction and classification of plant transcription factors, transcriptional regulators, and protein kinases. Mol Plant.

[14] Schultz J, Milpetz F, Bork P, Ponting CP (1998). SMART: a simple modular architecture research tool: Identification of signaling domains. Proc Natl Acad Sci.

[15] Aron MB, Lu S, Anderson JB, Farideh C, Derbyshire MK, Carol DWS, Fong JH, Geer LY, Geer RC, Gonzales NR (2011). CDD: a conserved domain database for the functional annotation of proteins. Nucleic Acids Res.

[16] Larkin MA, Blackshields G, Brown NP, Chenna R, McGettigan PA, Mcwilliam H, Valentin F, Wallace IM, Wilm A, Lopez R, Thompson JD, Gibson TJ, Higgins DG (2007). ClustalW and ClustalX version 2. Bioinformatics.

[17] Sudhir K, Glen S, Koichiro T (2016). MEGA7: molecular evolutionary genetics analysis version 7.0 for bigger datasets. Mol Biol Evol.

[18] Bailey TL, Mikael B, Buske FA, Martin F, Grant CE, Luca C, Ren J, Li WW, Noble WS (2009). MEME Suite: tools for motif discovery and searching. Nucleic Acids Res.

[19] Wang Y, Tang H, Debarry JD, Tan X, Li J, Wang X, Tae-Ho L, Jin H, Barry M, Guo H (2012). MCScanX: a toolkit for detection and evolutionary analysis of gene synteny and collinearity. Nucleic Acids Res.

[20] Chen C, Chen H, Zhang Y, Thomas HR, Xia R (2020). TBtools: an integrative toolkit developed for interactive analyses of big biological data. Mol Plant.

[21] Letunic I, Bork P (2019). Interactive Tree Of Life (iTOL) v4: recent updates and new developments. Nucleic Acids Res.

[22] Jin J, Zhang H, Kong L, Gao G, Luo J (2014). PlantTFDB 3.0: a portal for the functional and evolutionary study of plant transcription factors. Nucleic Acids Res.

[23] Purcell S, Neale B, Todd-Brown K, Thomas L, Ferreira M, Bender D, Maller J, Sklar P, Bakker P, Daly MJ (2007). PLINK: a tool set for whole-genome association and population-based linkage analyses. Am J Human Genet.

[24] Wang K, Mingyao L, Hakonarson H (2010). ANNOVAR: functional annotation of genetic variants from high-throughput sequencing data. Nucleic Acids Res.

[25] Skinner ME, Uzilov AV, Stein LD, Mungall CJ, Holmes IH (2009). JBrowse: a next-generation genome browser. Genome Res.

[26] Wuyun T, Zhu XC, Zhu GP, Zhao H. Extraction and refining technology of Siberian apricot kernel oil (CN 105950277 B), China, Patent. June, 11, 2019.

[27] Wuyun T, Jiang ZM, Zhu XC, Zhu XC, Zhao H. Production method of Siberian apricot kernel tofu ice cream (CN 105913934 B), China, Patent. June, 11, 2019.

[28] Wenjie D, David CN, Gerald HL, Brandon M, James IM (2007). ViroBLAST: a stand-alone BLAST web server for flexible queries of multiple databases and user's datasets. Bioinformatics.

[29] Tal G, Alan O, Jonathan S, Carson S (2017). heatmaply: an R package for creating interactive cluster heatmaps for online publishing. Bioinformatics.

