# Supplementary material for: AprGPD: the apricot genomic and phenotypic database
Source: Plant Methods. 2021 Sep 23;17:98. doi: 10.1186/s13007-021-00797-4 (PMC8461998; doi:10.1186/s13007-021-00797-4)
Supplement: Supplementary file 1 — Additional file 1: Table S1. Distribution of nine species in China. Figure S1. Pathways involved in flowering time. Figure S2. Phylogenetic tree of MIKC_MADS family. Figure S3. Gene structure and conserved motifs of MIKC_MADS family in P. sibirica (F106). Figure S4. Gene structure and conserved motifs of MIKC_MADS family in P. armeniaca (Sungold). Figure S5. Gene structure and conserved motifs of MIKC_MADS family in P. armeniaca × P. sibirica (Longwangmao). Figure S6. Chromosomal collinear of MIKC_MADS family in P. sibirica (F106). Figure S7. Chromosomal collinear of MIKC_MADS family in P. armeniaca (Sungold). Figure S8. Chromosomal collinear of MIKC_MADS family in P. armeniaca × P. sibirica (Longwangmao). Figure S9. Expression of MIKC_MADS family in P. sibirica (F106). (a) Expression of fruit development. (b) Expression of kernel development. Figure S10. Expression of fruit development of MIKC_MADS family in P. armeniaca (Sungold). Figure S11. Expression of MIKC_MADS family in P. armeniaca × P. sibirica (Longwangmao). (a) Expression of fruit development. (b) Expression of kernel development. [file 13007_2021_797_MOESM1_ESM.docx]

**Table S1 Distribution of nine species in China.**

**
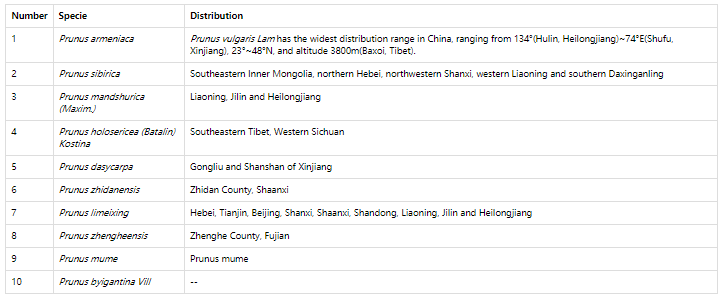
**


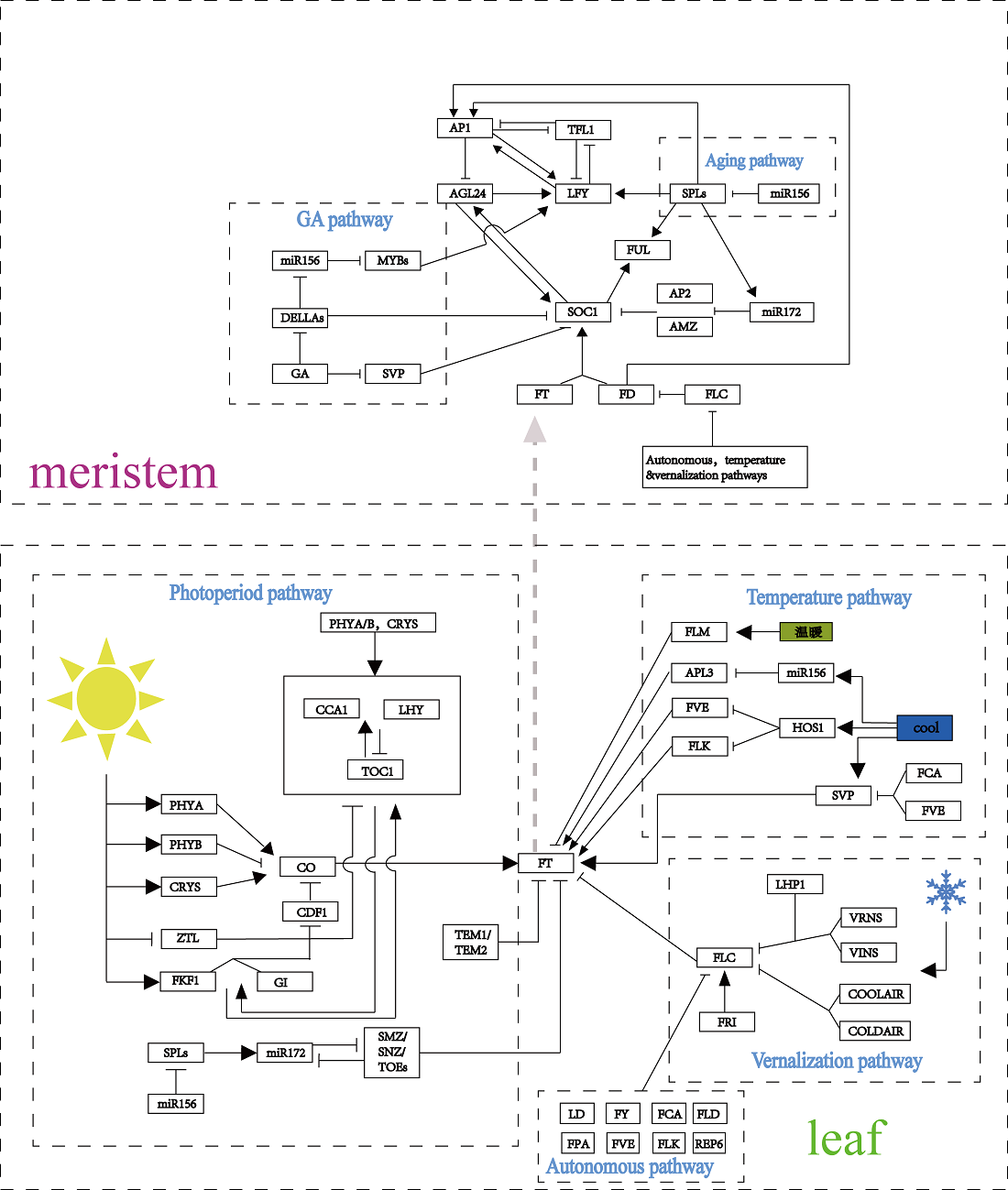


**Figure S1 Pathways involved in flowering time.**


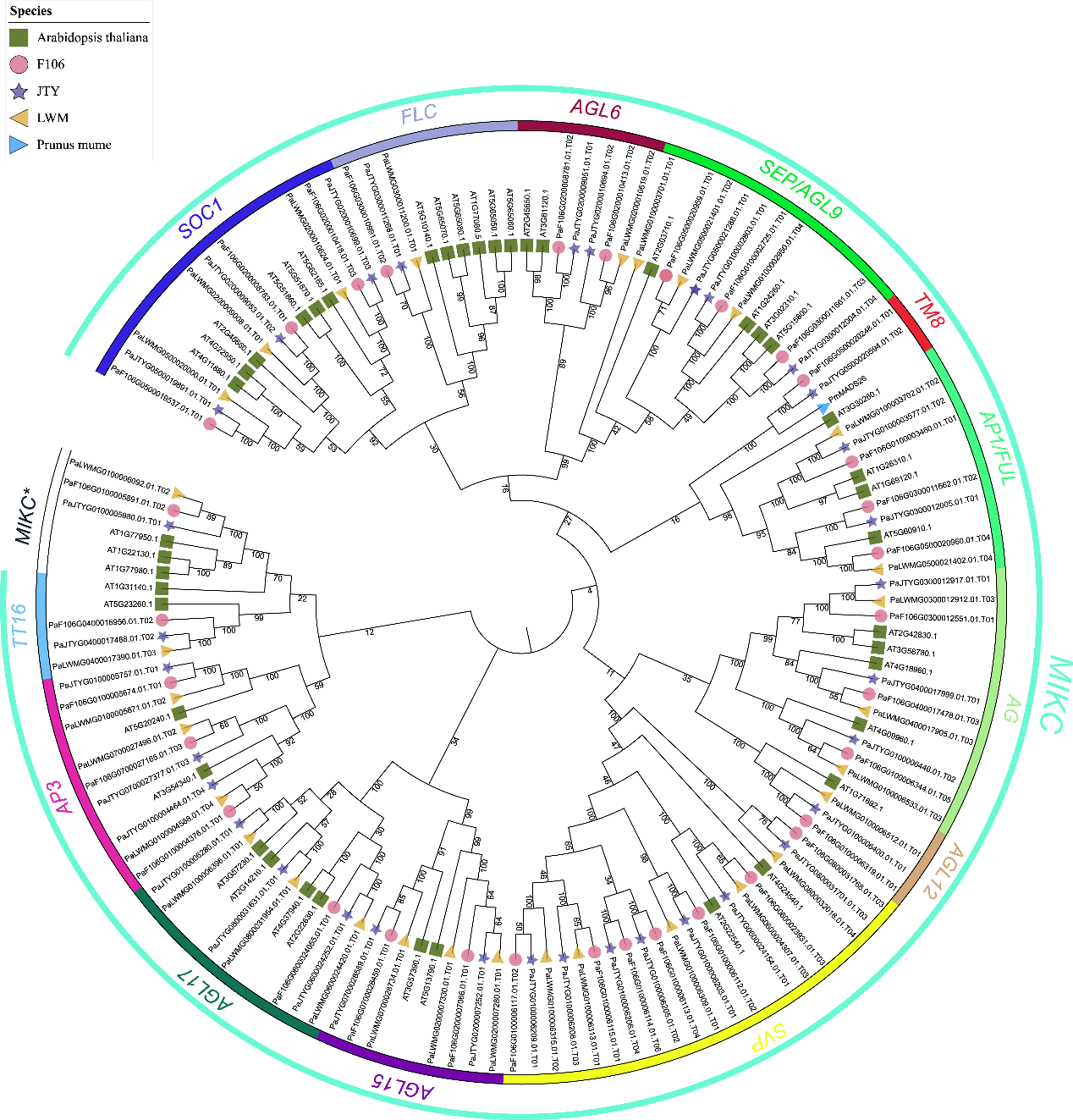


**Figure S2 Phylogenetic tree of MIKC_MADS family.**


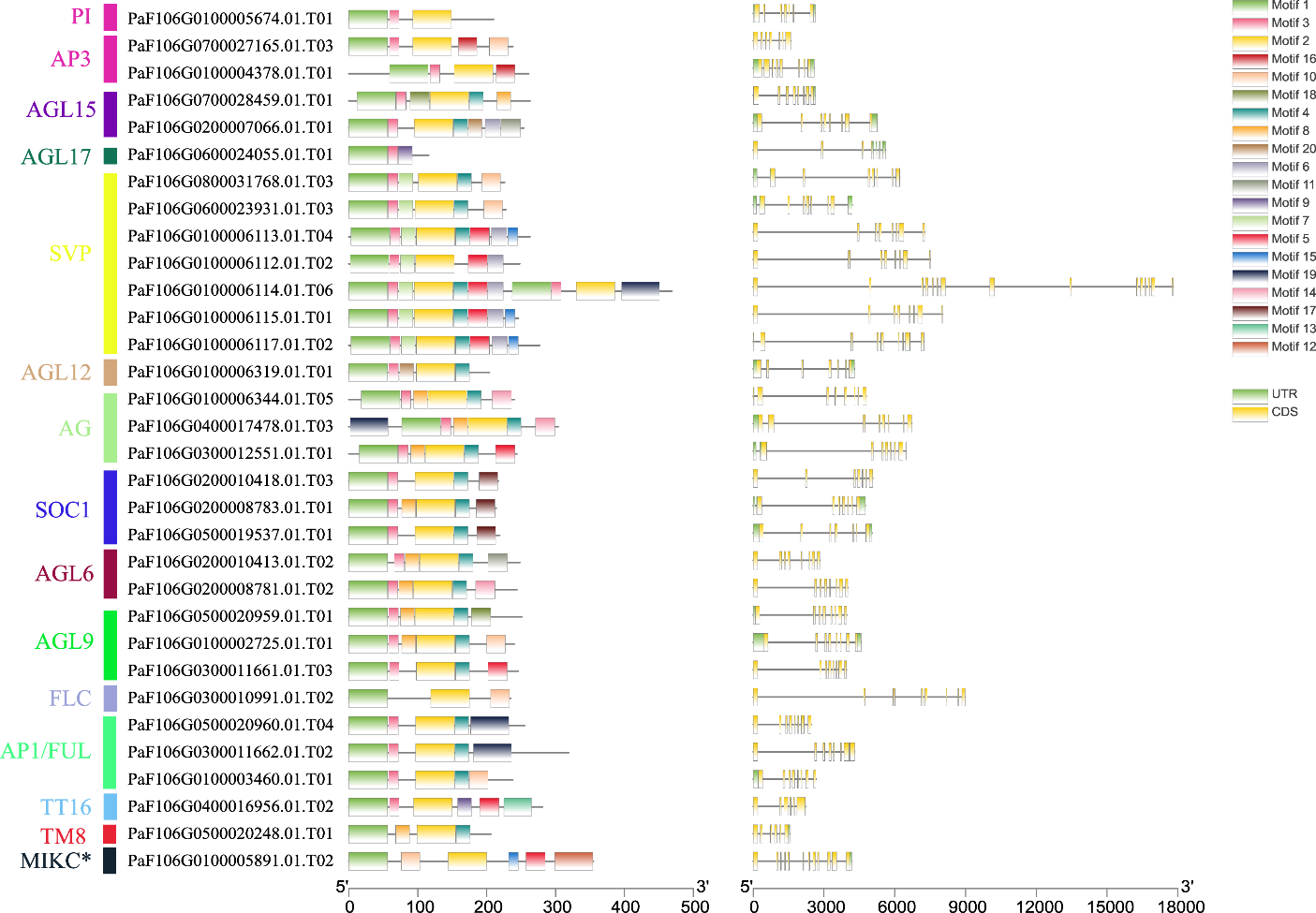


**Figure S3 Gene structure and conserved motifs of MIKC_MADS family in *P. sibirica* (F106).**


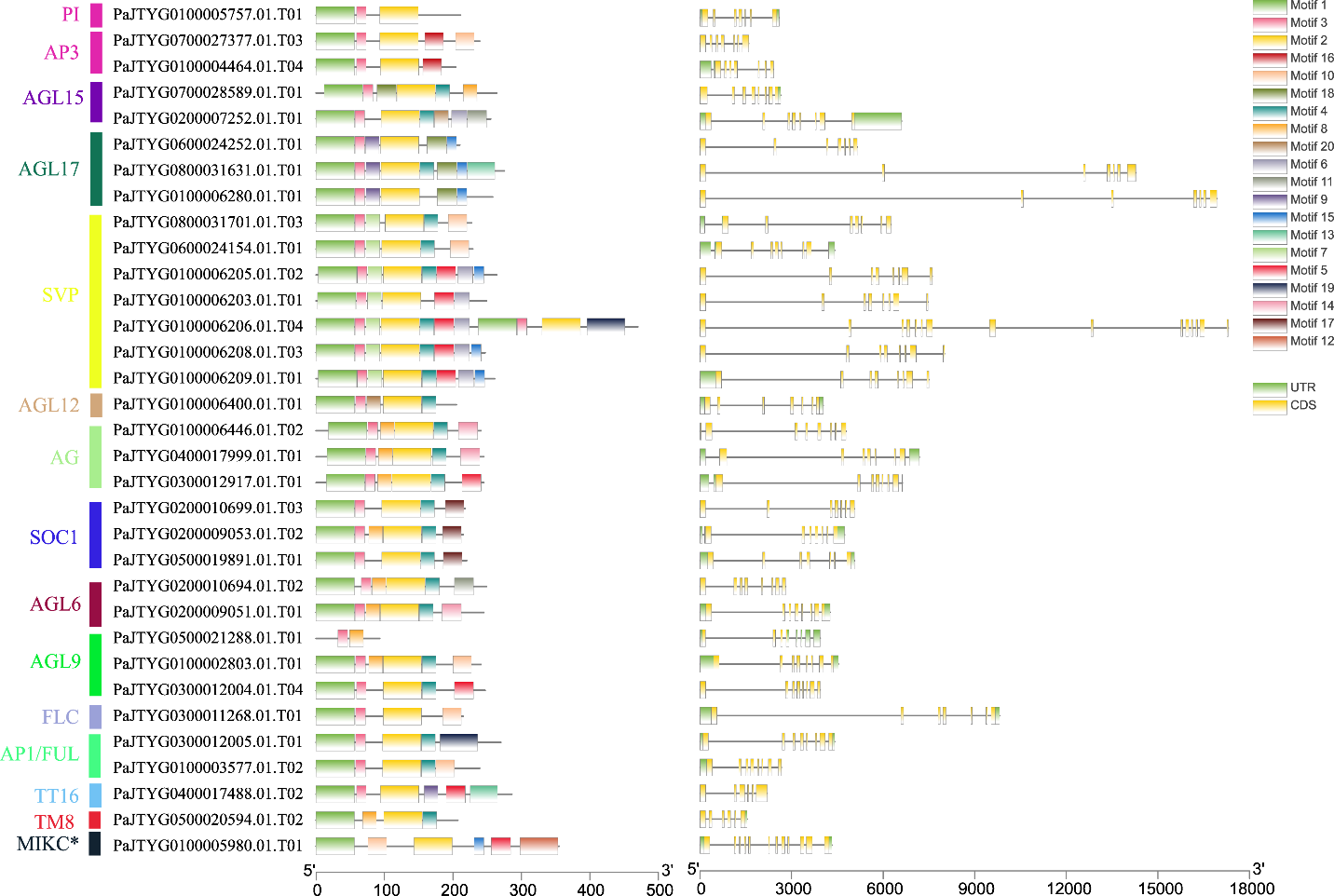


**Figure S4 Gene structure and conserved motifs of MIKC_MADS family in *P. armeniaca* (Sungold).**


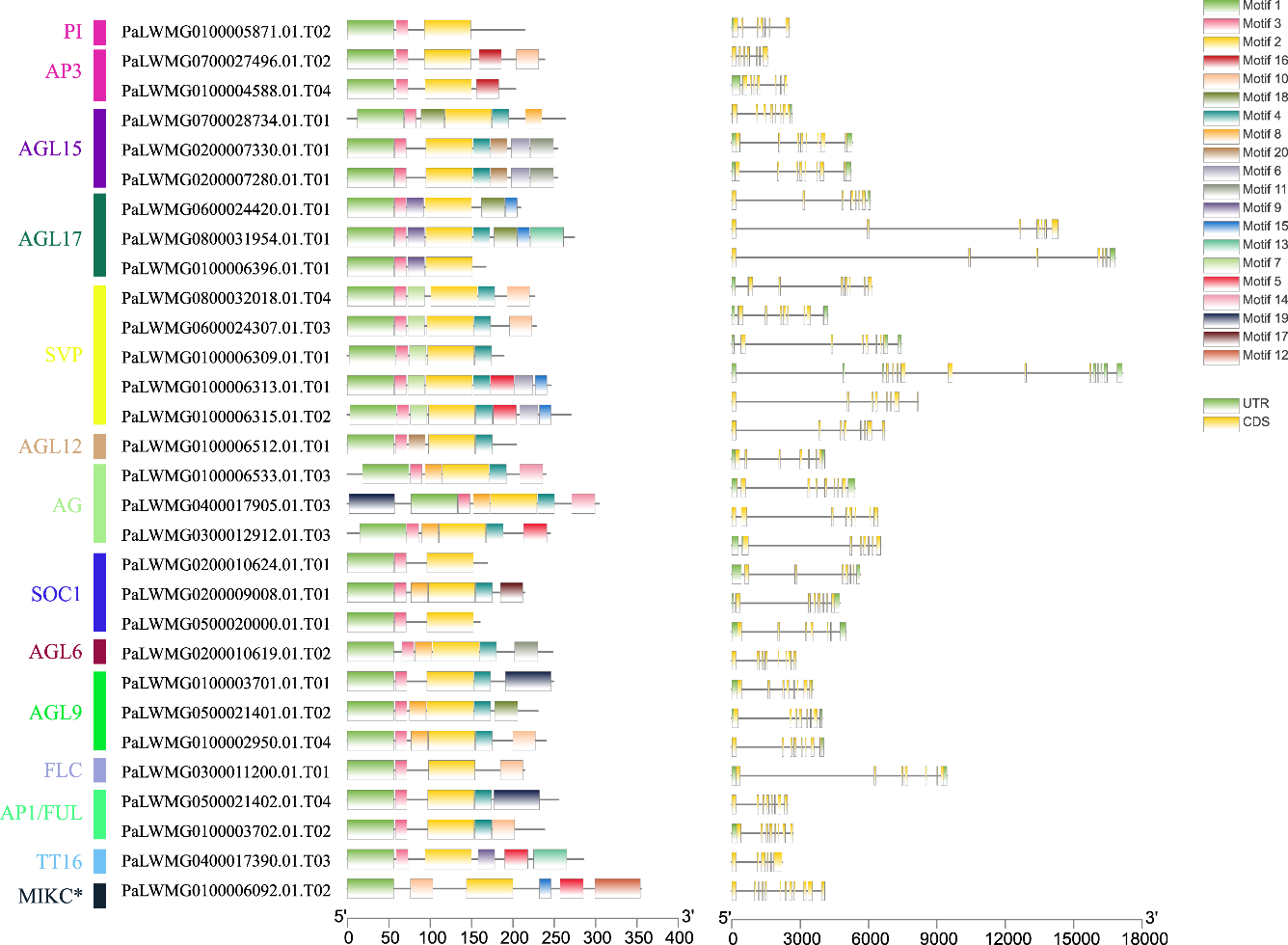


**Figure S5 Gene structure and conserved motifs of MIKC_MADS family in *P. armeniaca* × *P. sibirica* (Longwangmao).**


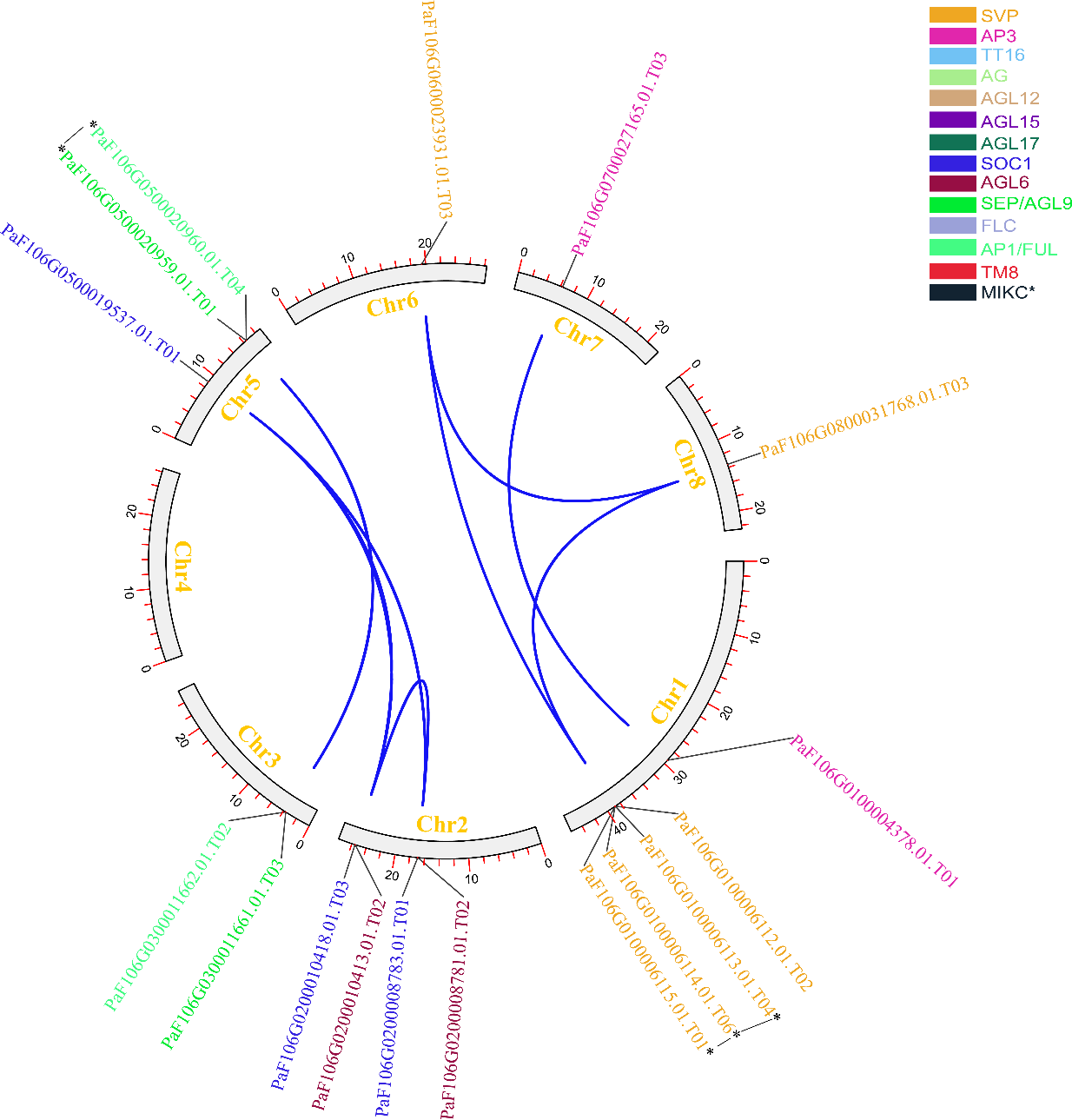


**Figure S6 Chromosomal** **collinear of MIKC_MADS family in *P. sibirica* (F106)**.


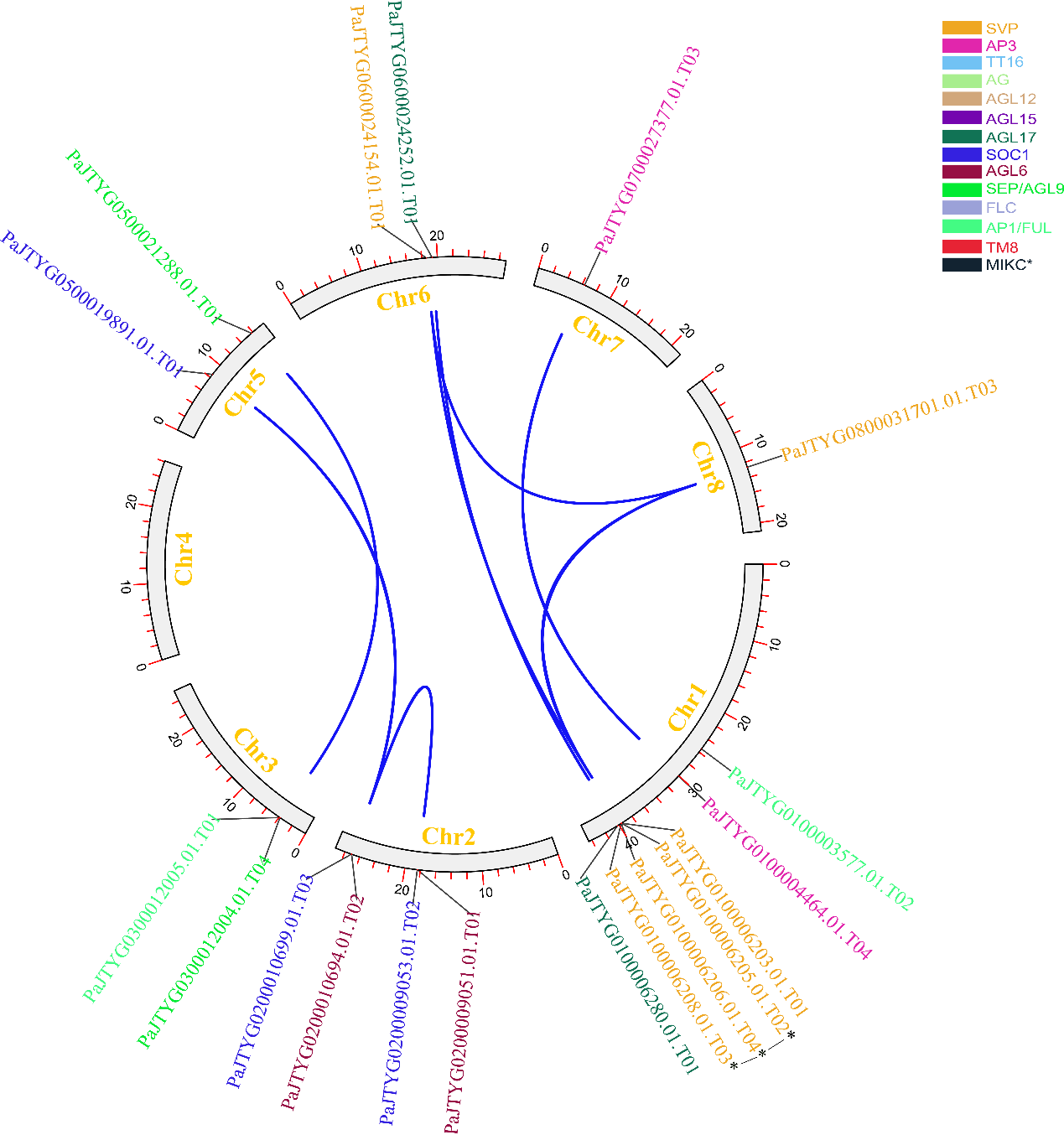


**Figure S7 Chromosomal** **collinear of MIKC_MADS family in *P. armeniaca* (Sungold).**


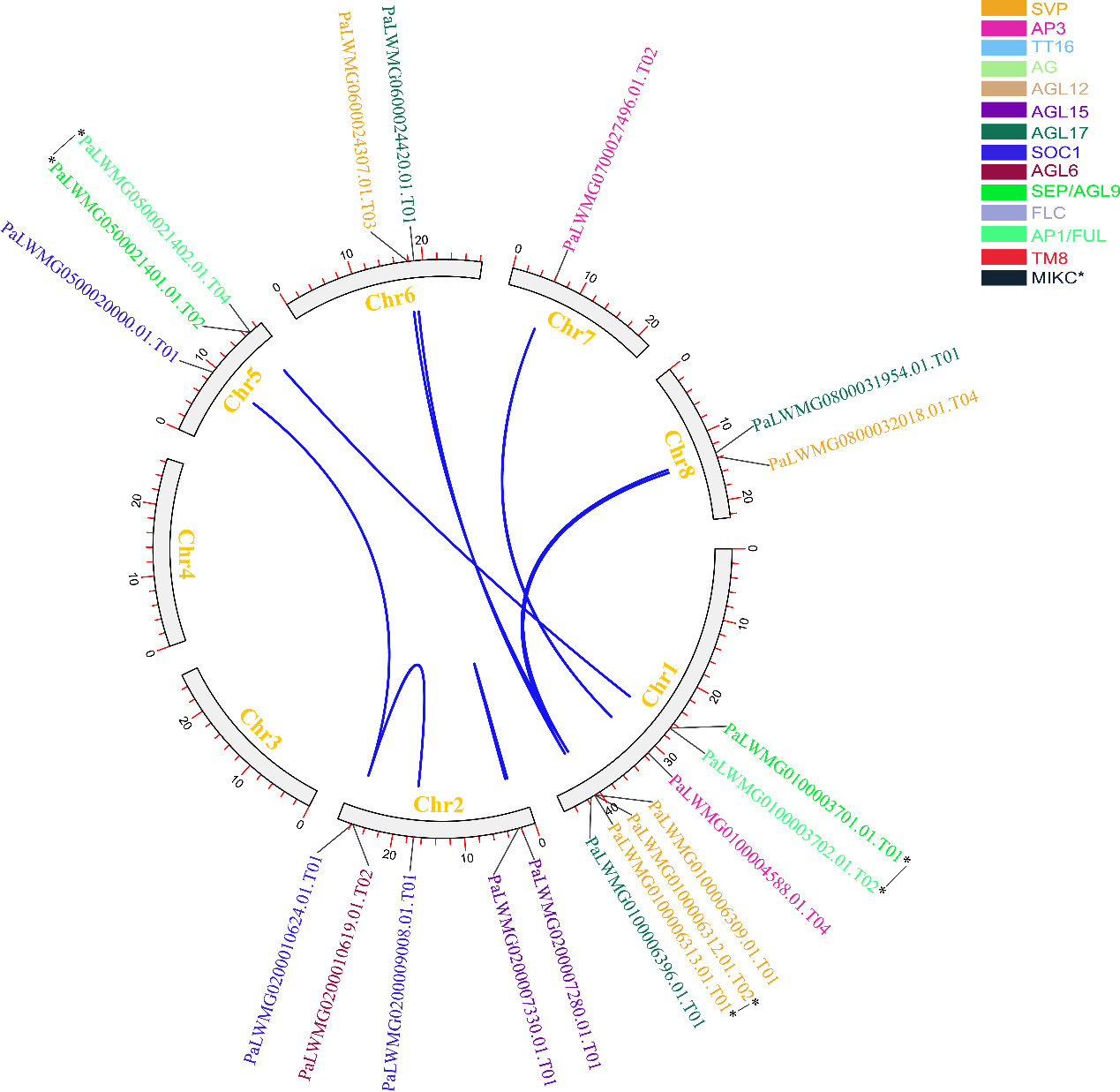


**Figure S8 Chromosomal** **collinear of MIKC_MADS family in *P. armeniaca* × *P. sibirica* (Longwangmao).**


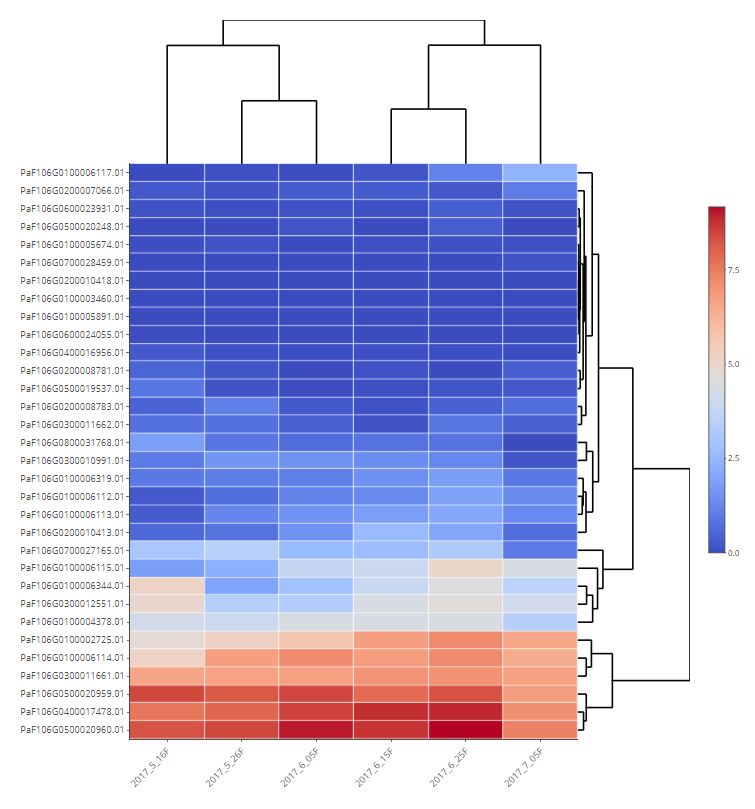

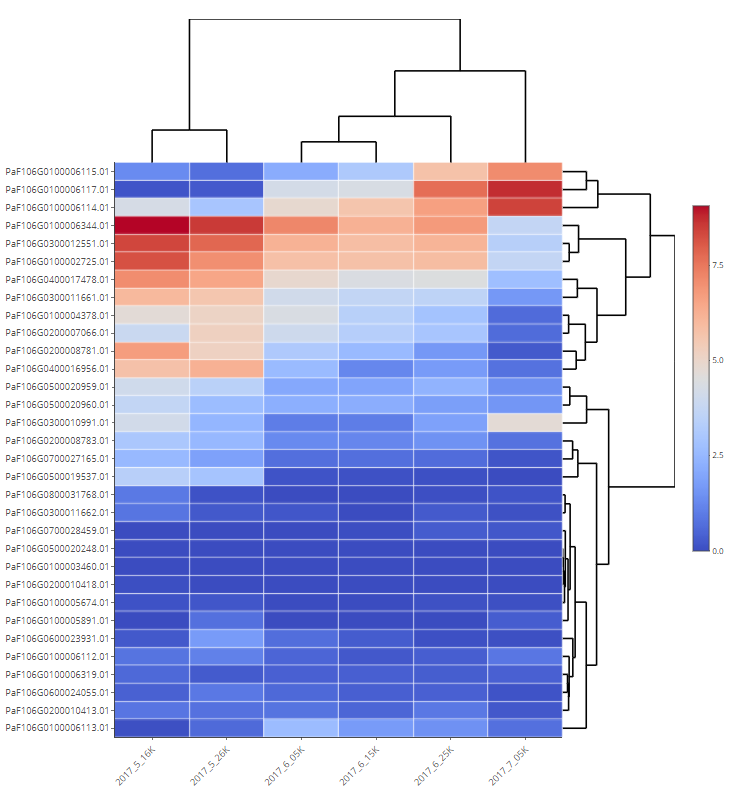


a b

**Figure S9 Expression of MIKC_MADS family in *P. sibirica* (F106).** (a) Expression of fruit development. (b) Expression of kernel development.


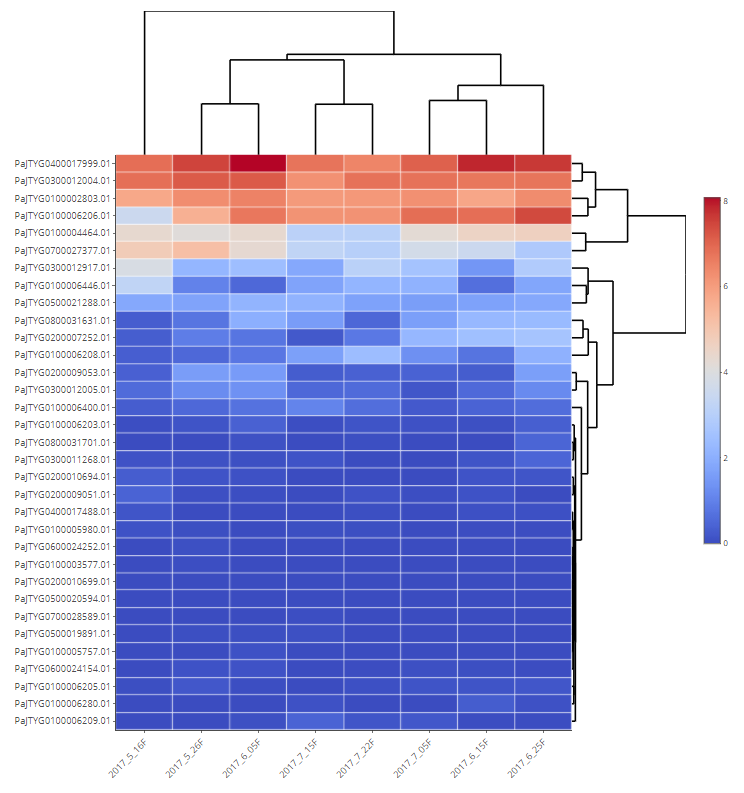


**Figure S10 Expression of fruit development of MIKC_MADS family in *P. armeniaca* (Sungold).**


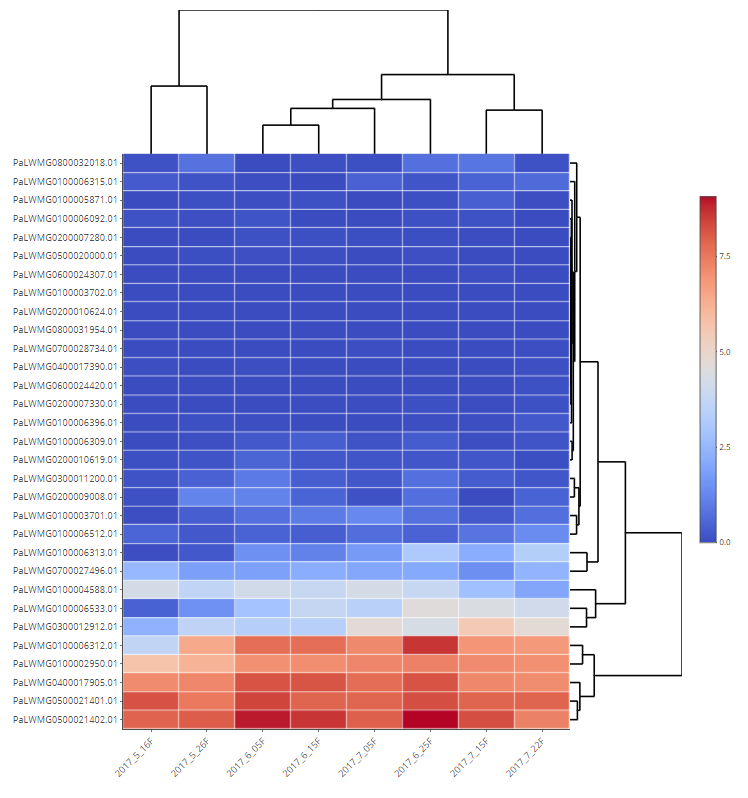

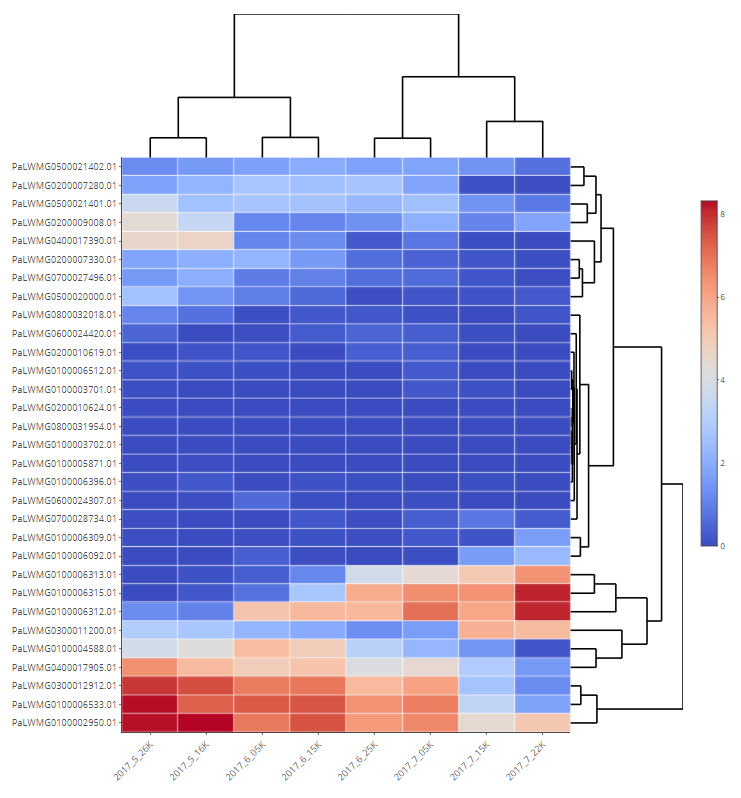


a b

**Figure S11 Expression of MIKC_MADS family in *P. armeniaca* × *P. sibirica* (Longwangmao).** (a) Expression of fruit development. (b) Expression of kernel development.
